# CD45 immunoaffinity depletion of vesicles from Jurkat T cells demonstrates that exosomes contain CD45: no evidence for a distinct exosome/HIV-1 budding pathway

**DOI:** 10.1186/1742-4690-5-64

**Published:** 2008-07-16

**Authors:** Lori V Coren, Teresa Shatzer, David E Ott

**Affiliations:** 1 AIDS and Cancer Virus Program, SAIC-Frederick, Inc., National Cancer Institute at Frederick, Frederick, Maryland, 21702-1201, USA

## Abstract

The presence of relatively high levels of cellular protein contamination in density-purified virion preparations is a confounding factor in biochemical analyses of HIV and SIV produced from hematopoietic cells. A major source of this contamination is from vesicles, either microvesicles or exosomes, that have similar physical properties as virions. Thus, these particles can not be removed by size or density fractionation. Although virions and vesicles have similar cellular protein compositions, CD45 is excluded from HIV-1 yet is present in vesicles produced from hematopoietic cells. By exploiting this finding, we have developed a CD45 immunoaffinity depletion procedure that removes vesicles from HIV-1 preparations. While this approach has been successfully applied to virion preparations from several different cell types, some groups have concluded that "exosomes" from certain T cell lines, specifically Jurkat, do not contain CD45. If this interpretation is correct, then these vesicles could not be removed by CD45 immunoaffinity depletion. Here we show that dense vesicles produced by Jurkat and SupT1/CCR5 cells contain CD45 and are efficiently removed from preparations by CD45-immunoaffinity depletion. Also, contaminating cellular proteins were removed from virion preparations produced by these lines. Previously, the absence of CD45 from both "exosomes" and virions has been used to support the so called Trojan exosome hypothesis, namely that HIV-1 is simply an exosome containing viral material. The presence of CD45 on vesicles, including exosomes, and its absence on virions argues against a specialized budding pathway that is shared by both exosomes and HIV-1.

## Findings

HIV-1 incorporates cellular proteins from the host cell during assembly and budding [1]. These proteins can provide important information about virus-cell interactions, yet biochemical analyses are greatly hindered by the presence of protein-laden vesicles in virion preparations, especially those produced by hematopoeitic cells. Because these vesicles co-purify with virions due to their similar size and density [2,3], they cannot be purified from virions using differences in physical properties alone. Vesicles can come from two sources: microvesicles that bud from the plasma membrane [4,5] and exosomes that form in late endosomal bodies and are released by exocytosis [6,7]. Therefore, we use the term vesicles to indicate the potential presence of both types of particles.

CD45 is abundantly expressed on the surface of hematopoeitic cells and their vesicles [8-11]. Nevertheless, CD45 protein is excluded from virions [9,11-13]. We have exploited this differential incorporation of CD45 to remove vesicles from virions by immunoaffinity depletion using anti-CD45-conjugated paramagnetic microbeads. While this technique has been used to produce high-purity virions for both biochemical and virus-cell interaction studies [12-14], it requires that vesicles contain sufficient amounts of CD45 for removal by the anti-CD45 beads. While we have consistently observed this in our experiments [12-14], two papers report that "exosomes" produced by Jurkat T cells, i.e. dense particles isolated from culture supernatants, do not contain CD45 [15,16], apparently excluding this protein during vesicle formation. If this were true and these "exosomes" are a distinct class of vesicle that do not contain CD45, then immunoaffinity depletion would not be able to remove vesicles from virus preparations isolated from cells that reputedly produce mostly "exosomes", e.g. Jurkat cells [15,16].

**Figure 1:**
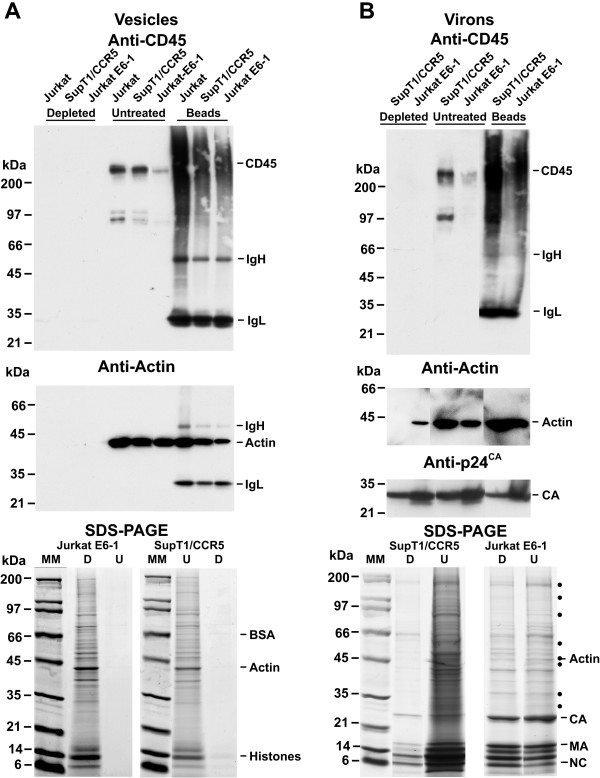
Immunoblots and SDS-PAGE gels of vesicles and virion samples. Immunoblots and SDS-PAGE gels of vesicle preparations (A) or virion preparations (B) (equal amounts by volume) isolated from cell cultures are presented. The samples are identified above their respective lanes. Antibody or antiserum used is indicated. Pertinent bands are identified at right of the blots. Cellular proteins reduced in depleted virion preparations are denoted in panel B with a dot at right. Cells were cultured in RPMI 1640 media with 2 mM L-glutamine, 100 U per ml penicillin, 100 μg per ml streptomycin and 10% vol/vol fetal bovine serum. CD45 immunoaffinity depletion was carried out using 100 μl of anti-CD45 paramagnetic microbeads (cat # 130-045-801, Miltenyi Biotec Inc.) that were washed in PBS and recovered by a magnetic separator (model MPC-S, Invitrogen, Inc.) twice before use. After an hour incubation at room temperature, the suspension was placed in a magnetic separator overnight at 4°C to capture the beads. The supernatant carefully removed from the beads and analyzed. Pan-specific CD45 antibody was obtained from BD-Transduction Laboratories, San Diego, CA, cat # 610266, Clone 69, IgG_1_. The pan-actin antibody was obtained from Amersham Biosciences, Arlington, IL, cat # N.350. CA antiserum was from the AIDS and Cancer Virus Program, NCI-Frederick, Goat # 81. SDS-PAGE gels were stained with by Coomassie brilliant blue to visualize proteins.

To determine whether these vesicles can be removed, we produced cell culture supernatants from uninfected Jurkat (gift of Kendall Smith, Cornell University, Ithaca, NY) and SupT1/CCR5 (gift of James Hoxie, University of Pennsylvania, Philadelphia, PA [17]) cells, two cell lines commonly used for the production of HIV-1. To confirm the Jurkat results we obtained the reference Jurkat E6-1 cell line [18] from the NIAID AIDS Reference Program. Vesicle preparations were produced from 50 mls of uninfected cell culture supernatants (material produced from a culture of 8 × 10^5 ^cells per ml for 48 hr) using the same 20% sucrose centrifugation procedure as used for virion preparations [19]. Half of the vesicle preparations (equivalent to 25 ml of supernatant) were subjected to CD45 immunoaffinity depletion. Equal amounts (by initial supernatant volume) of both depleted and untreated vesicles were examined by immunoblotting and SDS-PAGE analysis as previously described [20]. Immunoblotting with a pan-specific CD45 antibody did not detect any CD45 signal in the depleted samples, while there was a strong signal in both the untreated Jurkat and SupT1/CCR5 samples (Figure 1A). A somewhat weaker signal was detected in the untreated Jurkat E6-1 sample, presumably due to less CD45 on the vesicles. Nevertheless, this signal was also removed by depletion. The bead fractions for each sample showed an intense signal from the captured CD45.

Actin is an abundant component of vesicles and can be used as a marker for vesicular contamination in the absence of virus [21]. To assay for the presence of this protein, the blot was stripped of the CD45 signal and exposed to a pan-actin antibody. The results showed that, similar to the CD45 finding, actin was not detectable in the depleted samples, but was present as an intense band in all three untreated samples as well as the bead fractions (Figure 1A). Based on the intensities of the actin bands (Figure 1A), equal amounts of material were loaded for each vesicle preparation, confirming the lower levels of CD45 present on the Jurkat E6-1 vesicles. Yet, even this lower level of CD45 was sufficient for removal vesicles from the preparation.

To examine their overall protein composition, the samples were examined by SDS-PAGE gel electrophoresis. The depleted preparations contained only a few faint bands (data not shown), mostly corresponding to bovine serum proteins such as albumin, which are carried-over from the culture medium during the initial density preparation. To remove these medium-based contaminants and allow for a clearer assessment of protein content, vesicles were isolated from three separate harvests of SupT1/CCR5 and Jurkat E6-1 cell supernatants by two sequential density centrifugation steps. SDS-PAGE gel analysis of these preparations after CD45-depletion did not detect any protein (representative data in Figure 1A) except for faint histone bands in the SupT1/CCR5 samples, likely from DNA complexes that co-purify due to their density (>1.5 g/ml) [22]. In contrast, the untreated vesicle samples contained (Figure 1A) a wide range of proteins including actin and histones but no BSA. An additional set of vesicle preparations were produced from 90 ml of culture supernatants and half of each was CD45-depleted. The proteins in the resulting matched samples were then quantified by the Bio-Rad DC kit (Hercules, CA) using a BSA standard. Results of duplicate determinations from the three independent isolations showed that CD45 depletion effectively removed the vesicular proteins (95%, SD ± 3%, n = 7 from Jurkat E6-1 and 96% SD ± 2%, n = 6 from SupT1/CCR5). These results and those above demonstrate that CD45 immunoaffinity depletion removes the vesicle-associated proteins produced by these cells.

The purpose of CD45 immunoaffinity depletion is to remove contaminants from virus preparations. To demonstrate this on virions, we infected both SupT1/CCR5 and Jurkat E6-1 cell lines with a stock of HIV-1_NL4-3 _(MOI ~0.1). Following two washes, cells were cultured in medium for 1 week and then virus was prepared from a 2-day harvest by density centrifugation. Equal amounts of HIV-1 by initial supernatant volume from the infected SupT1/CCR5 (0.8 μg CA) and Jurkat E6-1 (2.2 μg CA) cell cultures were CD45 immunoaffinity depleted. Depleted and untreated samples were examined by CD45 immunoblot analysis. The results showed that depletion removed all detectable CD45 from the treated samples (Figure 1B). Staining the blot for actin revealed that nearly all of the actin was removed from the virus preparations. Some actin did persist in the Jurkat E6-1 sample, consistent with some actin remaining inside the virion as previously observed [21].

The presence of virus was revealed by stripping and staining the blot with capsid (CA) antiserum: the treated samples had somewhat less intense staining CA bands than the untreated material. Similarly, the bead fractions had CA signal, though at a lower intensity than either the treated or untreated samples, indicating that some CA was removed by depletion, likely due to virus/vesicle aggregates that are formed by pelleting during purification [12]. This artifact is not observed when supernatants are depleted before centrifugation [12,13].

The SDS-PAGE gel results showed that the HIV-1 preparations from the SupT1/CCR5 cells contained a large amount of cellular proteins compared with the Jurkat E6-1 preparation (Figure 1B). CD45 immunoaffinity depletion markedly removed the contamination from the SupT1/CCR5 preparation, demonstrating the efficacy of the procedure. Because the Jurkat E6-1 preparation was relatively free from contamination, the removal of vesicles was less dramatic. However, the intensities of several cellular protein bands, including actin (labeled with dots in Figure 1B) decreased after depletion. Together with the CD45 and actin blots, these results show that the CD45 immunoaffinity procedure can remove cellular proteins from these virion preparations.

Overall, our results show that vesicles isolated from Jurkat and SupT1/CCR5 cells, whether microvesicles or exosomes, contain sufficient amounts of CD45 to allow for removal by anti-CD45 paramagnetic microbeads. This finding is in contrast to the previous reports that concluded that T cell "exosomes" from uninfected cells do not contain CD45 [15,16]. Despite procedural differences, our preparations should have contained at least some, if not all of the vesicular species that the other groups examined. Furthermore, CD45 has been detected in vesicle preparations from monocyte-derived macrophages [9], a cell type thought to produce mostly exosomes [23], and these particles can be effectively removed by CD45 depletion [13]. A more plausible explanation for the difference is that we use a CD45 antibody that recognizes an epitope in the cytoplasmic domain that is shared among all forms of CD45 for detection, while the other groups used antibodies that recognize its variable extracellular portion [15,16], thus may not detect all forms of CD45.

Booth et al. have proposed that HIV-1 relies extensively, if not exclusively, on an exosome budding pathway for release from the cell that is distinct from that of other particles [16]. This model is part of the authors' Trojan exosome hypothesis [24] which posits that HIV-1 is simply an exosome that contains HIV-1 components. Part of the support for HIV-1 using an exosome budding pathway was the apparent absence of CD45 from both virions and exosomes, implying a common CD45-free budding mechanism [16]. Thus, our data provided here do not support this type of a distinct, specialized and shared release pathway for HIV-1 and exosomes.

While CD45 immunoaffinity depletion can remove contaminating vesicles from preparations, some rare particles might remain. Formally, productive infection itself might induce the production of vesicles that lack CD45, though this has not been observed. It is important to note that, absolute biochemical purity of virion preparations may not be practically attainable and analyses should be evaluated with this important caveat in mind.

## Competing interests

The authors declare that they have no competing interests.

## Authors' contributions

TS purified virion preparations, LC carried out the immunoblot and SDS-PAGE analysis, and DO infected and maintained cells, carried out the CD45 immunoaffinity depletion, planned the experiments, analyzed data, and wrote the manuscript.

## References

[B1] Ott DE (2008). Cellular proteins detected in HIV-1. Rev Med Virol.

[B2] Bess JW, Gorelick RJ, Bosche WJ, Henderson LE, Arthur LO (1997). Microvesicles are a source of contaminating cellular proteins found in purified HIV-1 preparations. Virology.

[B3] Gluschankof P, Mondor I, Gelderblom HR, Sattentau QJ (1997). Cell membrane vesicles are a major contaminant of gradient-enriched human immunodeficiency virus type-1 preparations. Virology.

[B4] Trams EB, Lauter CJ, Salem N, Heine U (1981). Exfoliation of membrane ecto-enzymes in the form of micro-vesicles.. Biochem Biophys Acta.

[B5] Heijnen HF, Schiel AE, Fijnheer R, Geuze HJ, Sixma JJ (1999). Activated platelets release two types of membrane vesicles: microvesicles by surface shedding and exosomes derived from exocytosis of multivesicular bodies and alpha-granules. Blood.

[B6] Pan BT, Teng K, Wu C, Adam M, Johnstone RM (1985). Electron microscopic evidence for externalization of the transferrin receptor in vesicular form in sheep reticulocytes. J Cell Biol.

[B7] Stoorvogel W, Kleijmeer MJ, Geuze HJ, Raposo G (2002). The biogenesis and functions of exosomes. Traffic.

[B8] Miguet L, Pacaud K, Felden C, Hugel B, Martinez MC, Freyssinet JM, Herbrecht R, Potier N, van Dorsselaer A, Mauvieux L (2006). Proteomic analysis of malignant lymphocyte membrane microparticles using double ionization coverage optimization. Proteomics.

[B9] Nguyen DG, Booth A, Gould SJ, Hildreth JE (2003). Evidence that HIV budding in primary macrophages occurs through the exosome release pathway. J Biol Chem.

[B10] Wubbolts R, Leckie RS, Veenhuizen PT, Schwarzmann G, Mobius W, Hoernschemeyer J, Slot JW, Geuze HJ, Stoorvogel W (2003). Proteomic and biochemical analyses of human B cell-derived exosomes. Potential implications for their function and multivesicular body formation. J Biol Chem.

[B11] Esser MT, Graham DR, Coren LV, Trubey CM, Bess JW, Arthur LO, Ott DE, Lifson JD (2001). Differential incorporation of CD45, CD80 (B7-1), CD86 (B7-2), and major histocompatibility complex class I and II molecules into human immunodeficiency virus type 1 virions and microvesicles: implications for viral pathogenesis and immune regulation. J Virol.

[B12] Trubey CM, Chertova E, Coren LV, Hilburn JM, Hixson CV, Nagashima K, Lifson JD, Ott DE (2003). Quantitation of HLA class II protein incorporated into human immunodeficiency type 1 virions purified by anti-CD45 immunoaffinity depletion of microvesicles. J Virol.

[B13] Chertova E, Chertov O, Coren LV, Roser JD, Trubey CM, Bess JW, Sowder RC, Barsov E, Hood BL, Fisher RJ, Nagashima K, Conrads TP, Veenstra TD, Lifson JD, Ott DE (2006). Proteomic and biochemical analysis of purified human immunodeficiency virus type 1 produced from infected monocyte-derived macrophages. J Virol.

[B14] Melar M, Ott DE, Hope TJ (2007). Physiological levels of virion-associated HIV-1 envelope induce coreceptor dependent calcium flux. J Virol.

[B15] Blanchard N, Lankar D, Faure F, Regnault A, Dumont C, Raposo G, Hivroz C (2002). TCR activation of human T cells induces the production of exosomes bearing the TCR/CD3/zeta complex. J Immunol.

[B16] Booth AM, Fang Y, Fallon JK, Yang JM, Hildreth JE, Gould SJ (2006). Exosomes and HIV Gag bud from endosome-like domains of the T cell plasma membrane. J Cell Biol.

[B17] Means RE, Matthews T, Hoxie JA, Malim MH, Kodama T, Desrosiers RC (2001). Ability of the V3 loop of simian immunodeficiency virus to serve as a target for antibody-mediated neutralization: correlation of neutralization sensitivity, growth in macrophages, and decreased dependence on CD4. J Virol.

[B18] Weiss A, Wiskocil RL, Stobo JD (1984). The role of T3 surface molecules in the activation of human T cells: a two-stimulus requirement for IL 2 production reflects events occurring at a pre-translational level. J Immunol.

[B19] Ott DE, Chertova EN, Busch LK, Coren LV, Gagliardi TD, Johnson DG (1999). Mutational analysis of the hydrophobic tail of the human immunodeficiency virus type 1 p6(Gag) protein produces a mutant that fails to package its envelope protein. J Virol.

[B20] Ott DE, Coren LV, Gagliardi TD, Nagashima K (2005). Heterologous late-domain sequences have various abilities to promote budding of human immunodeficiency virus type 1. J Virol.

[B21] Ott DE, Coren LV, Kane BP, Busch LK, Johnson DJ, Sowder II RC, Chertova EN, Arthur LO, Henderson LE (1996). Cytoskeletal proteins inside human immunodeficiency virus type 1 virions. J Virol.

[B22] Polisky B, McCarthy B (1975). Location of histones on simian virus 40 DNA. Proc Natl Acad Sci U S A.

[B23] Denzer K, Kleijmeer MJ, Heijnen HF, Stoorvogel W, Geuze HJ (2000). Exosome: from internal vesicle of the multivesicular body to intercellular signaling device. J Cell Sci.

[B24] Gould SJ, Booth AM, Hildreth JE (2003). The Trojan exosome hypothesis. Proc Natl Acad Sci U S A.

